# An Adaptation To Life In Acid Through A Novel Mevalonate Pathway

**DOI:** 10.1038/srep39737

**Published:** 2016-12-22

**Authors:** Jeffrey M. Vinokur, Matthew C. Cummins, Tyler P. Korman, James U. Bowie

**Affiliations:** 1 Department of Chemistry and Biochemistry, University of California, Los Angeles, CA, 90095, USA

## Abstract

Extreme acidophiles are capable of growth at pH values near zero. Sustaining life in acidic environments requires extensive adaptations of membranes, proton pumps, and DNA repair mechanisms. Here we describe an adaptation of a core biochemical pathway, the mevalonate pathway, in extreme acidophiles. Two previously known mevalonate pathways involve ATP dependent decarboxylation of either mevalonate 5-phosphate or mevalonate 5-pyrophosphate, in which a single enzyme carries out two essential steps: (1) phosphorylation of the mevalonate moiety at the 3-OH position and (2) subsequent decarboxylation. We now demonstrate that in extreme acidophiles, decarboxylation is carried out by two separate steps: previously identified enzymes generate mevalonate 3,5-bisphosphate and a new decarboxylase we describe here, mevalonate 3,5-bisphosphate decarboxylase, produces isopentenyl phosphate. Why use two enzymes in acidophiles when one enzyme provides both functionalities in all other organisms examined to date? We find that at low pH, the dual function enzyme, mevalonate 5-phosphate decarboxylase is unable to carry out the first phosphorylation step, yet retains its ability to perform decarboxylation. We therefore propose that extreme acidophiles had to replace the dual-purpose enzyme with two specialized enzymes to efficiently produce isoprenoids in extremely acidic environments.

Extremophiles are organisms capable of surviving in the harshest conditions on earth such as temperatures exceeding 120 °C in hydrothermal vents, salinity exceeding 5 M NaCl in evaporating lakes, and acidity below pH 0 in acid mine drainage^1^,^2^,^3^. The vast majority of extremophiles belong to the archaeal domain of life, having adapted to conditions prevalent on a primordial earth^4^. Growth in extremely acidic conditions is especially challenging as the organism must maintain a 100,000 fold H^+^ gradient across its membrane while allowing for the import and export of metabolites and other molecules^5^.

The lowest pH to support life so far recorded is pH −0.06 (1.2 M sulfuric acid) by *Picrophilus torridus*, a member of the archaeal order thermoplasmatales^6^. This unique order contains only 11 characterized organisms, all of which are extreme acidophiles capable of growth at pH 0.5 and below^6^,^7^,^8^,^9^. Thermoplasmatales have among the smallest genomes of any free living organism (<2 Mb), reversed membrane potentials, and all but *P. torridus* lack a cell wall entirely^5^. The first line of defense against acidity in thermoplasmatales is a highly impermeable lipid monolayer made of C40 tetra-ether lipids^10^. The C40 alkyl chains are made entirely from tandem repeats of 5-carbon isoprene units, and are connected to polar head groups through ether linkages^10^. Isoprenoid based lipids pack more tightly, making archaeal membranes less permeable to small molecules and the ether linkages impart acid stability^11^.

The 5-carbon precursor for all isoprenoids, isopentenyl pyrophosphate (IPP), is generated by the mevalonate pathway in eukaryotes, archaea, and some bacteria^12^. All known mevalonate pathways first produce (R)-mevalonate through the condensation of three acetyl-CoA molecules followed by a reduction step to yield mevalonate (Fig. 1). In eukaryotes, mevalonate is then phosphorylated twice at the 5-OH position to generate mevalonate 5-pyrophosphate, then decarboxylated to yield IPP (Fig. 1, black pathway)^13^. In most archaea however, mevalonate is phosphorylated once to make mevalonate 5-phosphate (M5P), then decarboxylated to isopentenyl phosphate (IP), and finally phosphorylated again to generate IPP (Fig. 1, blue pathway)^14^,^15^. In both the eukaryotic and classical archaeal pathways, decarboxylation is ATP-dependent and proceeds in two sequential steps via a single enzyme: (1) phosphorylation at the 3-OH position of the mevalonate moiety using ATP and (2) decarboxylation (Fig. 2)^16^. Thus, the decarboxylases are dual function enzymes. The phosphorylation step adds a phosphate group to the 3-OH position, which acts as a good leaving group and primes the molecule for decarboxylation^16^.

We and another group recently discovered two new enzymes in *Thermoplasma acidophilum*, mevalonate 3-kinase (EC 2.7.1.185) and mevalonate 3-phosphate 5-kinase (EC 2.7.1.186), whose sequential catalysis produces mevalonate 3,5-bisphosphate from mevalonate suggesting that there may be another route to produce isoprenoids besides the canonical archaeal pathway (Fig. 1, red pathway)^17^,^18^. However, the putative mevalonate 3,5-bisphosphate decarboxylase (MBD) remained unidentified to complete this alternative archaeal pathway. Here we report the identification of MBD from *T. acidophilum*, which produces IP through the ATP independent decarboxylation of mevalonate 3,5-bisphosphate (Fig. 2, reaction in brackets).

The new pathway is particularly odd, because the 3-OH phosphorylation and subsequent decarboxylation are carried out by two distinct enzymes even though both enzymes are structurally homologous to dual-function decarboxylases. Thus, it appears that the two enzymes evolved from a dual function decarboxylase, but became specialized. Why? We show that the new pathway is only present in extreme acidophiles, suggesting that low pH may require divergent activities. Indeed we find that the dual function mevalonate 5-phosphate decarboxylase (MMD) from *Roseiflexus castenholzii* is unable to carry out the kinase step at low pH, but retains decarboxylase activity. Thus, it is possible that a separate enzyme became specialized to handle the kinase function at low pH. This adaptation could have evolved through a duplication or horizontal transfer event, followed by specialization to support life in extremely acidic environments.

## Results

### Sequence homology suggests that Ta0893 is a decarboxylase

To search for mevalonate 3,5-bisphosphate decarboxylase (MBD) in *Thermoplasma acidophilum*, we identified proteins homologous to decarboxylases. Two proteins from the archeon *Thermoplasma acidophilum*, Ta1305 and Ta0893, were computationally annotated in the year 2000 as “mevalonate pyrophosphate decarboxylase”^9^. As reported recently, however, Ta1305 is actually mevalonate 3-kinase (EC 2.7.1.185)^17^. A structural homology model of Ta0893 made with PHYRE2 suggested significant similarity to mevalonate 5-pyrophosphate decarboxylase from yeast (PDB: 1FI4, confidence score of 100%) and contained the invariant Asp/Lys/Arg catalytic triad necessary for decarboxylation in the correct positions (Fig. 3)^19^,^20^,^21^. Unfortunately, Ta0893 consistently formed inclusion bodies when expressed in *E. coli* and no activity could be detected in extracts. As a result we set out to find the putative missing decarboxylase via direct purification from *T. acidophilum*.

### Identification of MBD activity in *T. acidophilum* lysate

Following growth of *Thermoplasma acidophilum*, we were able to detect MBD activity in crude lysate using mevalonate 3,5-bisphosphate as a substrate. To initially fractionate the lysate, we separated the *T. acidophilum* lysate by anion exchange chromatography using a HiTrap Q HP column. Ten fractions were collected and assayed for MBD activity^18^. As shown in Fig. 4A, fraction 7 showed the highest MBD activity, so we further separated fraction 7 in two side-by-side lanes on a native polyacrylamide gel. Coomassie blue staining (detection limit: ~5 ng) of one lane revealed 25 distinct bands. The adjacent gel lane (unstained) was then cut into 19 fragments (Fig. 4B), pulverized and assayed for MBD activity. As shown in Fig. 4C, MBD activity was detected in gel fragments 13–16. Each fragment was independently analyzed by NanoLC/MS/MS for protein identification. The results revealed that Ta0893 and 6 other proteins were present in all 4 gel fragments (Table 1), suggesting that one of them might be the missing decarboxylase.

### Ta0893 shows decarboxylase activity when truncated or co-expressed with chaperones

Identification of Ta0893 through mass spectrometry combined with clear homology motivated a larger effort to produce active, soluble Ta0893 in *E. coli*. Nevertheless, all attempts to refold Ta0893 from inclusion bodies failed after varying many parameters such as pH, temperature, salts, substrates, and screening using the Quickfold kit from Athena Enzyme Systems^22^. We were finally able to detect MBD activity in Ni-NTA eluates under two expression conditions in *E. coli*: (1) truncating Ta0893 by removing 30 AAs from the N-terminus and (2) co-expression of the full-length Ta0893 protein with *E. coli* chaperones, GroES/GroEL (Fig. 4D)^23^. We chose to truncate 30 AAs from the N-terminus of Ta0893 because this region was not present in the Ta0893 homolog from *Thermoplasma volcanium*, suggesting the first 30 AAs were not required for function. Size exclusion chromatography showed that the apparent molecular weight of MBD activity is 89±4 kDa in both recombinant Ta0893 co-expressed with chaperones as well as *T. acidophilum* lysate (Fig. 4E). The predicted molecular weight of a Ta0893 monomer is 46.3 kDa, suggesting that Ta0893 is a native homodimer. The finding that MBD activity in *E. coli* is dependent on Ta0893 expression and correlates with the same molecular weight as activity in *T. acidophilum* lysate, strongly suggests that Ta0893 is the missing MBD.

### Ta0893 produces isopentenyl phosphate

To confirm that recombinant Ta0893 produces isopentenyl phosphate (IP), we coupled MBD activity to IP kinase from *T. acidophilum* which specifically phosphorylates IP^24^. Robust ATP consumption was detected when IP kinase was added to a reaction containing Ta0893 pre-incubated with (R)-mevalonate 3,5-bisphosphate (Fig. 4F).

### Other extreme acidophiles also display MBD activity

While we confirmed that Ta0893 is a *bona fide* MBD, the lack of robust Ta0893 expression led us to seek close homologs better suited for recombinant expression to characterize the biochemical properties of MBD. A BLASTp search for homologs of Ta0893 reveals only 8 organisms containing an MBD homolog with greater than 30% sequence identity. Indeed, these 8 organisms represent all the sequenced thermoplasmatales. We cloned each MBD homolog (*Thermoplasma volcanium, Picrophilus torridus, Ferroplasma acidarmanus, Ferroplasma sp. Type II*, and *Acidiplasma sp. MBA-1*), into *E. coli* expression vectors. Using standard expression conditions, we observed activity in Ni-NTA eluates for the *T. volcanium and P. torridus* MBD homologs (Fig. 5). MBD homologs from *Ferroplasma* and *Acidiplasma* formed inclusion bodies under all tested expression conditions.

### The specialized mevalonate pathway is unique to thermoplasmatales

A BLASTp search using MBD from *T. acidophilum* (Ta0893) shows strong sequence identity for all 8 sequenced thermoplasmatales. This includes *Acidiplasma sp. MBA-1* (68%), *Acidiplasma aeolicum* (68%), *Acidiplasma cupricumulans* (68%), *Thermoplasma volcanium* (63%), *Ferroplasma sp. Type II* (50%), *Ferroplasma acidarmanus* (48%), and *Picrophilus torridus* (40%) (Fig. 6A). The next strongest alignments (29%) after thermoplasmatales are proteins from eubacteria of the phylogenetic family chloroflexaceae. Indeed, the chlorflexaceae *Roseiflexus castenholzii* is known to contain a *bona fide* MMD of the canonical archaeal mevalonate pathway^14^. Sequence alignment of *T. acidophilum* MBD against classical mevalonate pathway decarboxylases shows retention of the invariant Asp/Lys/Arg catalytic triad required for decarboxylation, however MBDs are unique in that they are missing both nearly invariant ATP binding residues (Fig. 6B)^19^,^25^,^26^,^27^. Indeed, unlike all other mevalonate pathway decarboxylases, MBD does not require ATP. MBD’s complement, mevalonate 3-kinase (Ta1305) has equally strong homologs (39–67% ID) in all 8 thermoplasmatales yet has no detectable homologs in any other organism, suggesting that this pathway is unique to thermoplasmatales.

### Biochemical characterization of MBD from *Picrophilus torridus*

Since the *P. torridus* MBD showed robust expression in *E. coli*, we chose to purify this enzyme for further characterization (Fig. 7A) and compare it to the classical mevalonate 5-phosphate decarboxylase (MMD) from *Roseiflexus castenholzii*^14^. *Roseiflexus castenholzii* is a member of the chloroflexaceae family whose MMDs are most closely related to MBDs of *Thermoplasmatales* and are therefore the closest known evolutionary precursors. Purified *P. torridus* MBD showed clear specificity for mevalonate 3,5-bisphosphate and no detectable decarboxylase activity on mevalonate, mevalonate 3-phosphate, mevalonate 5-phosphate or mevalonate 5-pyrophosphate (Fig. 7B, black bar). *P. torridus* MBD worked optimally at 70 °C and a pH around 5 where it had a k_cat_ of approximately 7.0 s^−1^ with respect to (R)-mevalonate 3,5-bisphosphate (Supplemental Figs 1–3). This k_cat_ value is comparable to *R. castenholzii* MMD (1.7 ± 0.1 s^−1^)^14^. Interestingly, *R. castenholzii* MMD was also active on mevalonate 3,5-bisphosphate when supplied with ADP as a co-factor (Fig. 7B, blue bars). We were unable to accurately determine the K_m_ for MBD since it was still at V_max_ when assayed at the detection limit of our GC-FID assay (30 μM).

### MMD loses kinase function at low pH

We characterized the previously un-reported activity of MMD on its reaction intermediate (mevalonate 3,5-bisphosphate) and compared it to its native substrate. Interestingly, at low pH, MMD completely loses its ability to convert mevalonate 5-phosphate to IP (its native reaction), but continues to decarboxylate mevalonate 3,5-bisphosphate, effectively becoming a MBD at low pH because it cannot perform the first kinase step (Fig. 8). The fact that decarboxylase activity remains intact suggests that low pH does not cause global inactivation or unfolding of the enzyme. These results suggest that low pH requires specialization to provide a kinase function. The adaptation could come in the form of adjustments to the normal MMD or the evolution of a new kinase.

## Discussion

**Figure 1:**
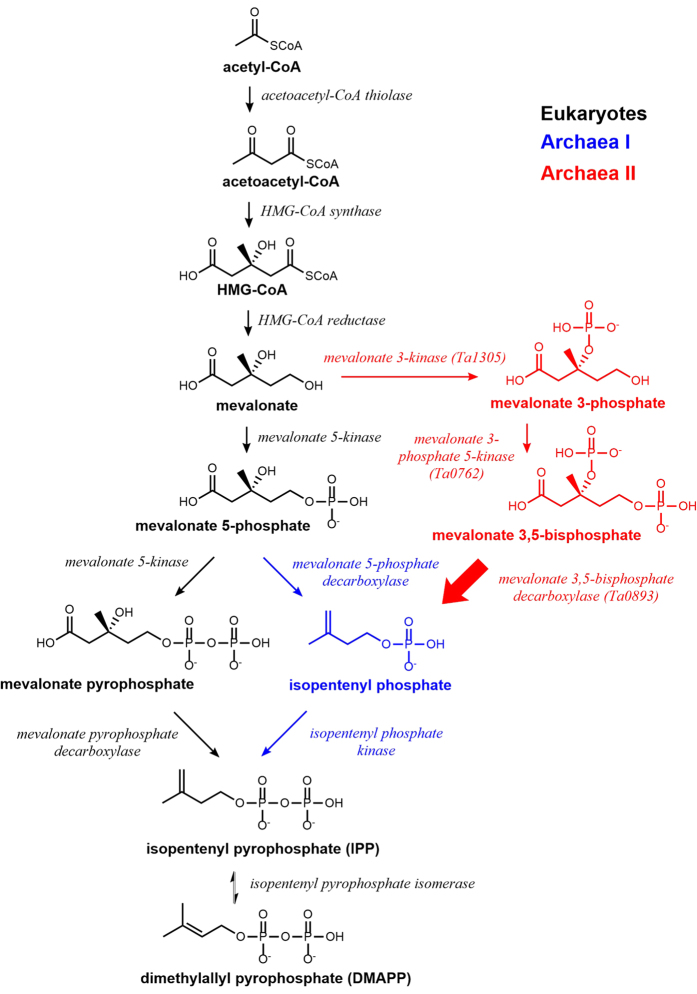
Mevalonate pathways. Eukaryotes use the pathway shown in black while most archaea use the pathway shown in blue. Here we have identified mevalonate 3,5-bisphosphate decarboxylase (bold arrow) which confirms a third route present in extreme acidophiles (red).

**Figure 3:**
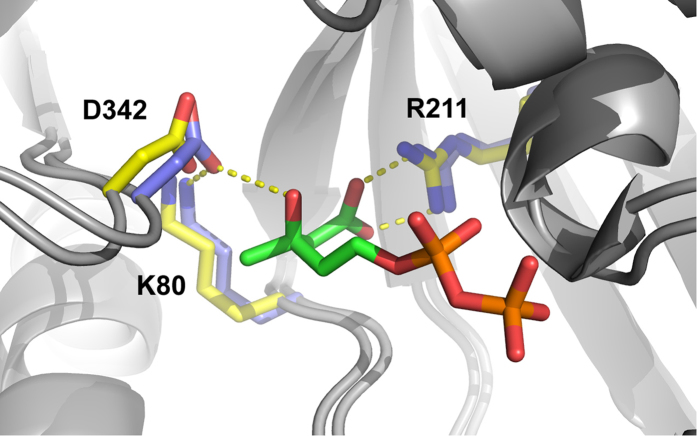
Ta0893 possesses key residues for decarboxylation. A PHYRE model of Ta0893 is overlayed with mevalonate pyrophosphate decarboxylase from *S. epidermidis* containing bound mevalonate 5-pyrophosphate (PDB: 4DU7). Numbered Ta0893 residues are highlighted in yellow and mevalonate pyrophosphate decarboxylase residues are shown in blue.

**Figure 4:**
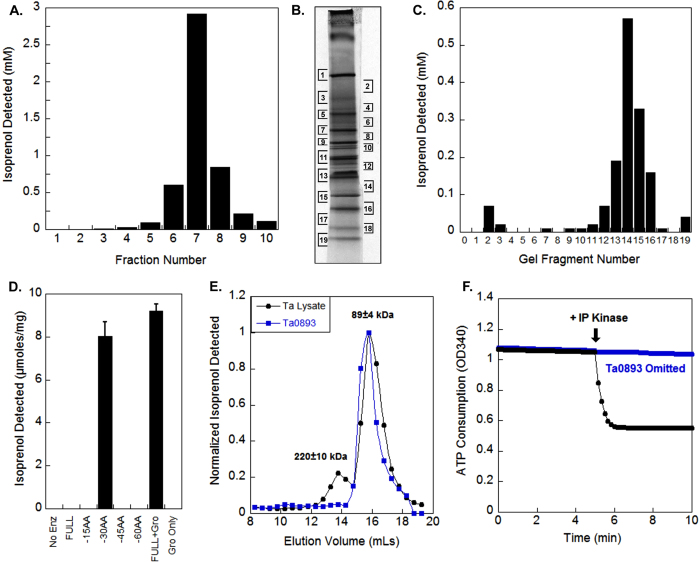
Purification and identification of MBD. (**A**) MBD activity in anion exchange fractions of a *T. acidophilum* lysate. (**B**) Native gel separation of fraction 7 from anion exchange. (**C**) The native gel was cut into 19 fragments, pulverized, and tested for MBD activity. (**D**) Units are in umoles/mg total protein. MBD activity in crude extracts with various N-terminal truncations of Ta0893 expressed in *E. coli* or when the full length protein was co-expressed with GroEL/ES (Gro). (**E**) *T. acidophilum* lysate (black) and recombinant Ta0893 co-expressed with GroEL/ES (blue) were separated on a Superdex S200 gel filtration column. The MBD activity peaked at the same volume in both samples, consistent with the identification of Ta0893 as the native MBD. (**F**) Addition of IP kinase to the product of Ta0893 causes rapid consumption of ATP (black) suggesting that Ta0893 produces IP. A negative control omitted Ta0893 (blue).

**Figure 5:**
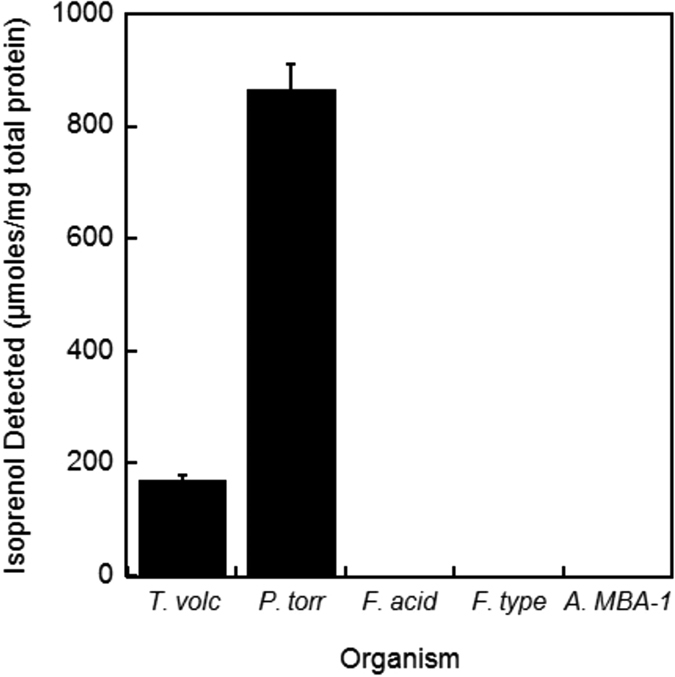
Activity of Ta0893 homologs. Five Ta0893 homologs were his-tag purified and incubated with mevalonate 3,5-bisphosphate to detect MBD activity. Homologs from *Ferroplasma* and *Acidiplasma* formed inclusion bodies under all expression conditions. The MBD homolog from Acidiplasma sp. MBA-1 has the identical amino acid sequence as A. aeolicum and is 99.7% identical (1AA substitution) to A. cupricumulans. We cloned the MBA-1 homolog to represent all 3 species of the Acidiplasma genus. NCBI accession numbers: T. acidophilum (WP_010901303.1), T volcanium (WP_010916684.1), P. torridus (WP_010917040.1), F. acidarmanus (WP_009887850.1), F. sp. Type II (EQB73519.1), and A. sp. MBA-1 (WP_048100791.1).

The novel decarboxylase reported here demonstrates a unique mevalonate pathway in *T. acidophilum*. While the classical archaeal pathway phosphorylates mevalonate at the 5-OH position to yield mevalonate 5-phosphate, and then uses MMD to produce IP in an ATP dependent reaction, the *T. acidophilum* pathway produces the same end product, but uses a different set of enzymes and metabolites^17^. The pathway in *T. acidophilum* phosphorylates mevalonate at the 3-OH position and the 5-OH position sequentially by two distinct enzymes to yield mevalonate 3,5-bisphosphate. This is followed by the action of MBD which carries out ATP independent decarboxylation to produce IP. Both archaeal pathways use IP kinase to produce IPP (Fig. 1, blue and red routes)^24^.

Localization of this unique pathway to the most acid tolerant organisms on earth suggests that the pathway may confer an evolutionary advantage in extremely acidic environments. *P. torridus* has an internal pH of 4.6 while *T. acidophilum* and *F. acidarmanus* have an internal pH of 5.5 and 5.6 respectively^28^,^29^,^30^. We observe that at pH 4.3, the conversion of mevalonate 5-phosphate by MMD stops completely, while the decarboxylation activity of mevalonate 3,5-bisphosphate by MMD remains intact. We propose that the common ancestor of thermoplasmatales also had a low internal pH and the classical decarboxylation reaction was inefficient, which would have applied evolutionary pressure to adapt. One possible way to adapt would be to find a way to make both the kinase and decarboxylation steps more effective at low pH. The fact that such a seemingly simple adaptation did not occur, suggests that it may be difficult to accomplish. Interestingly, a different evolutionary pathway was chosen by thermoplasmatales and two separate enzymes developed. We propose a model in which a horizontal transfer or a gene duplication event placed two MMD enzymes into the common ancestor and over time these two enzymes became specialized^9^,^31^. One lost its decarboxylase function to become a mevalonate 3-kinase, and the second lost its kinase function to become a dedicated decarboxylase (MBD). These two specialized enzymes were more efficient at low pH than a single dual-function enzyme. One possible explanation for the kinase function of *R. castenholzii* MMD stopping while decarboxylation continues at low pH could be the acidity perturbing the protonation state of an active site residue that deprotonates the 3-OH group of mevalonate and triggers nucleophilic attack on the γ-phosphate of ATP.

One peculiar observation is that the two specialized functions do not act in tandem, but are one step removed, with mevalonate 3-phosphate 5-kinase acting between the 3-OH phosphorylation of mevalonate and the decarboxylation by MBD. The reason for this is unclear.

Decarboxylation in the mevalonate pathway has been extensively studied in the eukaryotic homolog, mevalonate 5-pyrophosphate decarboxylase, which converts mevalonate 5-pyrophosphate to IPP in an ATP-dependent manner^16^,^21^,^32^,^33^,^34^,^35^. The mechanism was previously thought to occur via a single step in which the phosphorylation of the 3-OH position leads to an unstable and transient intermediate that rapidly decomposes into IPP, CO_2_ and PO_4_^26^,^27^. Our previous identification of mevalonate 3,5-bisphosphate as a stable metabolite suggested that decarboxylation is not spontaneous and must be carried out by enzyme catalysis^17^,^36^. The identification of MBD provides further support for the two-step model since *T. acidophilum* carries out the reactions through two entirely separate enzymes. Furthermore, we show that MMD from *Roseiflexus castenholzii* can accept its intermediate, mevalonate 3,5-bisphosphate, and carry out the second step of its dual function independently of the first step. These results strongly indicate that decarboxylation is catalyzed, not spontaneous, and that the ATP dependent decarboxylases operate via a two-step mechanism.

**Figure 6:**
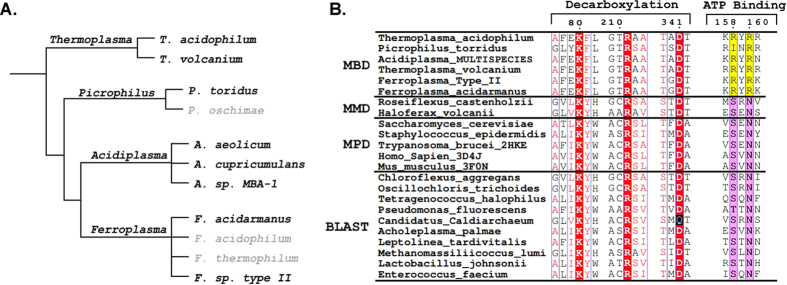
Phylogeny and Sequence Alignment. (**A**). A phylogenetic tree of thermoplasmatales based on 16 S rRNA. Organisms with no available DNA sequences are shown in grey. Environmental samples were excluded for clarity. (**B**) Mevalonate 3,5-bisphosphate decarboxylase homologs representing all 8 sequenced thermoplasmatales were aligned with mevalonate 5-phosphate decarboxylases (MMD), mevalonate 5-pyrophosphate decarboxylases (MPD), and the top Ta0893 BLASTp hits after thermoplasmatales. MBDs retain the Asp/Lys/Arg catalytic triad required for decarboxylation (red), but are missing both ATP binding residues (purple).

**Figure 7:**
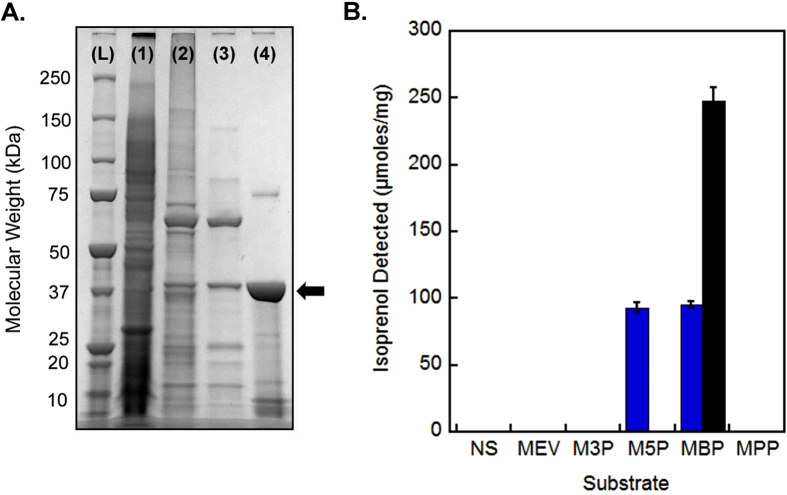
Purification of *P. torridus* MBD and Substrate Specificity. (**A**) An SDS-PAGE gel stained with Coomassie blue. Lanes: (L) ladder, (1) crude *E. coli* lysate, (2) Ni-NTA eluate, (3) supernatant after heating for 2 hrs at 60 °C, (4) flow through from a Q HP anion exchange column. Yield was 1 mg purified *P. torridus* MBD from 32 L of culture. (**B**). *P. torridus* MBD (black) and *R. castenholzii* mevalonate 5-phosphate decarboxylase (blue) were assayed via our GC-FID decarboxylase assay after incubation with the following substrates for 1 hour: (MEV) mevalonate, (M3P) mevalonate 3-phosphate, (M5P) mevalonate 5-phosphate, (MBP) mevalonate 3,5-bisphosphate, (MPP) mevalonate 5-pyrophosphate and (NS) no substrate.

**Figure 8:**
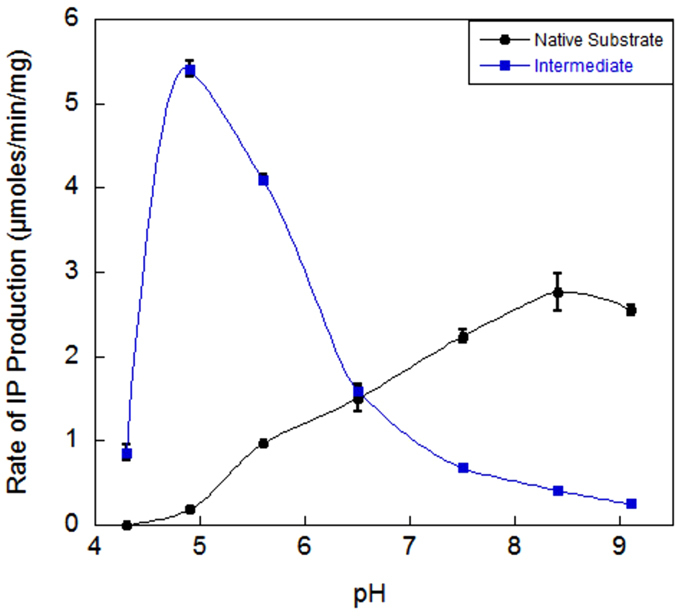
pH dependence of activity of *R. castenholzii* MMD with native substrate (mevalonate 5-phosphate) or intermediate (mevalonate 3,5-bisphosphate). Mevalonate 5-phosphate decarboxylase was assayed at 50 °C over a pH range of 4.3-9.1 with 10 mM mevalonate 5-phosphate (black) or 10 mM mevalonate 3,5-bisphosphate (blue).

One unexpected finding of our study is that MMD from *Roseiflexus castenholzii* requires ADP as a co-factor to decarboxylate mevalonate 3,5-bisphosphate. We propose that ADP binding is necessary to form the active enzyme-substrate complex. Structural analysis of mevalonate 5-pyrophosphate decarboxylase from *Staphylococcus epidermidis* showed that ATP binding triggers two flexible loops to close over the substrates in the active site pocket^32^. We suggest that ADP is necessary as a co-factor to mimic the presence of the native substrates (mevalonate 5-phosphate and ATP), which would trigger loop closure and form the active complex. After decarboxylation, the loops would presumably transition to an open state and release IP, ADP, CO_2_ and PO_4_. Sequence alignment with mevalonate pyrophosphate decarboxylases shows that MBD does not contain two nearly invariant amino acids that bind the adenine ring of ATP (Fig. 6B)^37^. Presumably, the specialization of MBD to become a dedicated decarboxylase involved the loss of its ADP requirement.

In summary, we have identified and characterized mevalonate 3,5-bisphosphate decarboxylase, a novel enzyme which produces isopentenyl phosphate and completes the unique mevalonate pathway of extreme acidophiles comprising the archaeal order thermoplasmatales. We also report that the two steps of the mevalonate 5-phosphate decarboxylase mechanism can be separated as demonstrated by the robust activity of MMD directly on its intermediate, mevalonate 3,5-bisphosphate. Indeed, at pH values representative of the *P. torridus* cytoplasm (~4.6), MMD loses its kinase function completely and becomes a decarboxylase only. We propose that thermoplasmatales adapted their mevalonate pathway by replacing MMD with two specialized enzymes in order to produce isoprenoids in extremely acidic environments.

## Methods

### Materials

*E. coli* BL21(DE3) Gold (Agilent) was grown in Miller LB media (Fisher) for both cloning and expression of recombinant proteins. Plasmid pET28a(+) was purchased from Novagen and pBB541 encoding GroEL/GroES was obtained from Addgene^23^. Ni-NTA resin was purchased from Qiagen. Native gels were purchased from Expedeon. All other chemicals were purchased from Sigma-Aldrich unless otherwise noted.

### Thermoplasma acidophilum growth

*Thermoplasma acidophilum* was obtained as a live culture from NITE Biological Resource Center (Tokyo, Japan). The organism was grown in NBRC medium 280 at 60 °C to OD_600_ = 0.5. Cells were harvested by centrifugation at 5000 × g. 2 g of wet cell pellet (4 L culture) were resuspended in 20 mL of 50 mM sodium phosphate buffer [pH 6.5]. The cells were chilled on ice for 30 min and then lysed by sonication. A cell-free lysate was obtained after centrifugation at 30,000 × g.

### Anion exchange chromatography

Initial separation of the *T. acidophilum* lysate was adapted from the procedure of another research group^18^. In brief, 10 mL of *T. acidophilum* lysate was applied to a 1 mL HiTrap Q HP anion exchange column (strong quaternary ammonium anion exchanger) pre-equilibrated with 50 mM sodium phosphate buffer [pH 6.5], connected to an AKTA FPLC system. The column was washed with 20 mL of 50 mM sodium phosphate buffer [pH 6.5] followed by a gradient from 0 to 1 M NaCl over 20 min and 2 mL fractions were collected. A flow rate of 1 mL/min was used for all steps.

### Native gels

20 μL of the most active fraction from anion exchange chromatography was loaded into two side-by-side wells of a 20% native polyacrylamide gel (Expedeon). The gel was developed by running towards the anode at 40 V for 50 min followed by 150 V for 16 hrs in a cold room (4 °C). One lane was stained with Expedeon InstantBlue protein stain. The unstained lane was cut into 19 fragments using the stained lane as a reference. Each gel fragment was pulverized by sonication in 500 μL of 50 mM sodium phosphate buffer [pH 6.5] containing 500 mM NaCl. 50 μL of the resulting slurry was used for the GC-FID assay below.

### GC-FID Decarboxylase Assay

A 50 μL sample of chromatography fraction was added to a 150 μL reaction mixture consisting of 50 mM sodium phosphate buffer [pH 6.5], 500 mM NaCl, 10 mM (R)-mevalonate, 20 mM ATP, 5 mM MgCl_2_, 10 μg mevalonate 3-kinase (Ta1305), and 10 μg mevalonate-3-phosphate-5-kinase (Ta0762). The reaction was incubated for 24 hours at 60 °C. Any generated isopentenyl phosphate was then hydrolyzed into isoprenol and free phosphate by adding 100 μL of 1 M bis-tris propane [pH 9.0], followed by 30 U of alkaline phosphatase from bovine intestinal mucosa. After incubation at 37 °C for 2 hours, the reaction was extracted with 200 μL hexanes. 5 μL of the hexanes layer was injected into a HP5890 Series II Gas Chromatograph (flame ionization detector) connected to a HP-INNOWAX column (0.320 mm × 30 m, Agilent). The carrier gas was helium with a flow rate of 5 mL/min. Initial oven temperature was set to 70 °C for 2 min, followed by a ramp of 20 °C/min for 1 min, and finally a ramp at 50 °C/min to a final temperature of 200 °C, which was held for 1 min. The inlet was kept at 250 °C and the detector at 330 °C. Isoprenol eluted at 2.98 min and the sample concentration was determined by comparison to a standard. All GC-FID samples were prepared in duplicate.

### Mass spectrometry

The four native gel bands from the stained lane corresponding to the highest activities were excised and submitted to ProtTech Inc. for independent analysis via NanoLC/MS/MS. The following steps were carried out by Protech: Peptides were digested in-gel using sequencing grade modified trypsin (Promega) in 100 mM ammonium bicarbonate [pH 8.5] buffer. DTT and iodoacetamide were added for reduction and alkylation of cysteine residues. The digested peptides were extracted with acetonitrile, dried using a Thermo SpeedVac, then redissolved in 2% acetonitrile, 97.5% water, and 0.5% formic acid. Peptides were separated using a high pressure liquid chromatography system (HPLC) fitted with a reversed-phase C18 column (75 μM ID × 8 cm). Samples eluted from the HPLC column were directly ionized by electrospray ionization and analyzed by an ion trap mass spectrometer (LCQ DECA XP Plus, Thermo). MS/MS spectra were acquired via low energy collision induced dissociation. The collected mass spectrometric data were searched against the NCBI protein database using ProtTech’s ProtQuest software. Peptides were reported with a mass range of 550 to 1800 Da and a signal to noise ratio greater than or equal to 5.

### General Cloning

All genes were codon optimized and synthesized by IDT. Each gene was inserted between the NdeI and XhoI sites of the pET28a(+) vector, which allowed for the addition of an N-terminal 6xHis tag. Genes were synthesized with an extra 25 base-pairs complementary to the pET28a(+) vector at the NdeI and XhoI sites. A standard Gibson method was used to assemble all constructs by mixing 30 ng of synthesized DNA, with 10 ng of pET28a(+) digested with NdeI and XhoI and 7.5 μL of Gibson assembly mix^38^. After incubation at 50 °C for 2 hrs, 5 μL was used to transform *E. coli* BL21(DE3) Gold and transformants were selected on LB-agar plates containing 50 μg/mL kanamycin.

### Expression and Purification

Protein expression and purification was carried out as described previously^17^. In brief, 1 L of LB media was inoculated with 5 mL of *E. coli* overnight starter culture with 50 μg/mL kanamycin and/or 100 μg/mL spectinomycin as needed. The cells were grown to an OD_600_ of 0.5–1.0 and induced at 37 °C with 1.0 mM IPTG. After 18 hours, cells were pelleted, resuspended in 5 mL of buffer A (50 mM bis-tris propane [pH 7.5] buffer, 100 mM NaCl, 10 mM imidizole), lysed by sonication, and cell debris removed by centrifugation at 30,000 × g for 20 min. The lysate was mixed with 3 mL of a Ni-NTA slurry and incubated for 15 min at 4 °C with gentle mixing. The lysate mixture was packed into a column and the Ni-NTA beads were washed 3 times with 20 mL of buffer A. Protein was eluted with 4 mL of buffer A containing 250 mM imidazole. Co-expression of GroEL/GroES was achieved by the addition of plasmid pBB541 (addgene) to the expression strain^23^. For biochemical characterization, *P. torridus* MBD was further purified by heating the eluate at 60 °C for 2 hrs to precipitate native *E. coli* proteins followed by one passage through a HiTrap Q HP anion exchange column to remove negatively charged proteins.

### Coupled Enzyme Assay

The MBD product was confirmed to be IP via coupling to IP kinase. A 100 μL reaction containing 25 mM bis-tris propane buffer [pH 6.5], 5 mM (R)-mevalonate, 10 mM ATP, 5 mM KCl, 5 mM MgCl_2_, 5 μg mevalonate 3-kinase (Ta1305), 5 μg mevalonate 3-phosphate 5-kinase (Ta0893), and 50 μL Ta0893 Ni-NTA eluate (0.5 mg/mL total protein) was incubated for 24 hours at 60 °C. To measure ATP consumption, we added 1 μL coupling enzyme mix (lactate dehydrogenase and pyruvate kinase mix from rabbit muscle, Sigma), 15 mM PEP, and titrated in NADH via 0.5 mM increments to bring the OD_340_ to 1.0. We then recorded the OD_340_ over 10 min on a SpectraMax M5 microplate reader. 5 μg IP kinase from *T. acidophilum* (Ta0103) was added at the 5 min mark. A negative control replaced Ta0893 eluate with water.

### Biochemical characterization

The optimal pH for *P. torridus* MBD was determined in 0.5 pH unit increments. Mevalonate 3,5-bisphosphate was enzymatically generated by mixing 10 mM (R)-mevalonate with 20 mM ATP, 5 mM MgCl_2_, 100 mM NaCl, 10 μg mevalonate 3-kinase (Ta1305), and 10 μg mevalonate-3-phosphate-5-kinase (Ta0762). After 1 hour at 60 °C, 150 μL of this mixture was combined with 50 μL of 1 M sodium acetate (for pH 3.5–5.5) or bis-tris propane buffer (for pH 6.5–8.0). pH adjustments were made using HCl and NaOH and confirmed using a micro pH electrode (Hanna Inst. 1083B). The reaction was initiated by the addition of 0.1 μg *P. torridus* MBD and incubated at 60 °C for 1 hour followed by transferring the vials into boiling water for 1 min to stop the reaction. Analysis was carried out via GC-FID as described above. The optimal temperature was determined in the same manner with pH held constant at 5.5. The data points for optimum pH and temperature were confirmed to be initial rates by quenching a control reaction (pH 5.5, 60 °C) at 30, 60, 90, and 120 min as shown in Supplemental Fig. S3. Substrate specificity was tested by replacing (R)-mevalonate with commercially available (R)-mevalonate 5-phosphate, or (R)-mevalonate 5-pyrophosphate. Activity on mevalonate-3-phosphate was tested by omitting Ta0762. Kinetic characterization of *P. torridus* MBD were carried out at pH 5.5 and 70 °C using the GC-FID assay with 3 ng enzyme over a range of 0.03 to 5 mM (*R*)-mevalonate 3,5-bisphosphate. 50 μL aliquots of this reaction were quenched at 0, 20, 40, and 60 minutes by boiling for 1 min. The enzyme was at V_max_ for all substrate concentrations from 5 mM to the detection limit (30 μM). All characterization experiments were carried out in duplicate.

### Size exclusion chromatography

To estimate the size of the protein complex that causes MBD activity, we mixed 100 μL of *T. acidophilum* cell-free lysate or 100 μL recombinant Ta0893 with 100 μL of Biorad gel filtration standard and injected the mixture directly onto a Superdex S200 10/300 GL gel filtration column. The column was equilibrated in 50 mM sodium phosphate buffer [pH 6.5] and 500 mM NaCl at a flow rate of 0.5 mL/min. 0.5 mL fractions were collected and subjected to our GC-FID decarboxylase assay. Fitting the activity profile to a standard curve made from the internal standards allowed us to estimate the size of the MBD protein complex.

### Sequence Alignment

Ta0893 was subjected to a standard BLASTP search^39^. The resulting output was aligned using clustal omega with default parameters^37^. Mevalonate pyrophosphate decarboxylases from the PDB were also included in the alignment. The output was rendered with Espript 3.0 and edited with Macromedia Fireworks 2004 to highlight regions of interest^40^.

### Analysis of MMD activity at low pH

*Roseliflexus castenholzii* mevalonate 5-phosphate decarboxylase was assayed over a pH range of 4.3–9.1 with enzymatically generated substrates. Mevalonate 5-phosphate was produced by mixing 10 mM (R)-mevalonate with 20 mM ATP, 10 mM MgCl2, 100 mM NaCl and 20 μg mevalonate 5-kinase (M5K) from *Methanosarcina mazei*^41^. Mevalonate 3,5-bisphosphate was generated in an identical manner except that M5K was replaced with 10 μg of mevalonate 3-kinase (Ta1305) and 10 μg mevalonate 3-phosphate 5-kinase (Ta0762). After production of the substrates for 4 hr at 37 °C and 60 °C respectively, we mixed 150 μL of each substrate mixture with 50 μL of 1 M acetate buffer (pH 4.3–5.6) or bis-tris propane buffer (6.5–9.1). A micro pH electrode (Hanna Inst. 1083B) was used to confirm the final pH. 0.5 μg of *R. castenholzii* MMD was then added to each reaction. After incubation at 50 °C for 1 hour, we stopped the reaction by immersing the vials in boiling water for 1 min and analyzed it via the GC-FID protocol above. This experiment was carried out in duplicate.

## Additional Information

**How to cite this article**: Vinokur, J. M. *et al*. An Adaptation To Life In Acid Through A Novel Mevalonate Pathway. *Sci. Rep.* **6**, 39737; doi: 10.1038/srep39737 (2016).

**Publisher's note:** Springer Nature remains neutral with regard to jurisdictional claims in published maps and institutional affiliations.

## Supplementary Material

Supplementary Information

## Figures and Tables

**Figure 2:**
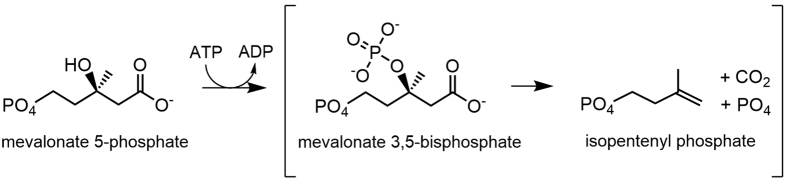
The reaction scheme. Mevalonate 5-phosphate decarboxylase of the classical archaeal pathway employs a two-step mechanism: (1) phosphorylation using ATP and (2) decarboxylation. The enzyme reported here, mevalonate 3,5-bisphosphate decarboxylase, carries out only the second step shown in brackets.

**Table 1:** Proteins identified in gel fragments with MBD activity.

Hits	Prot. Mass (kDa)	Peptides	Protein Annotation	NCBI Accession	Rel. Abund.
1	37.7	107	Deoxyhypusine Synthase (Ta0356)	WP_010900784	83.5%
2	44.4	9	S-adenosylmethionine Synth. (Ta0059)	*WP_010900487*	8.4%
3	46.4	4	Hypothetical Protein (Ta0893)	WP_010901303	0.4%
4	51.1	4	Glutamine Synthetase (Ta1498)	WP_010901897	0.6%
5	55.0	1	Hypothetical Protein (Ta0203)	WP_010900630	0.2%
6	45.0	1	Hypothetical Protein (Ta0204)	WP_010900631	0.1%
7	35.5	1	DNA Repair Protein RadA (Ta1104)	WP_010901514	0.0%

NanoLC/MS/MS identified seven proteins which were present in the entire region of MBD activity (gel fragments 13–16, Fig. 4B). The relative abundance is reported for fragment 14.
