# Supplementary material for: An Adaptation To Life In Acid Through A Novel Mevalonate Pathway
Source: Sci Rep. 2016 Dec 22;6:39737. doi: 10.1038/srep39737 (PMC5177888; doi:10.1038/srep39737)
Supplement: Supplementary Information [file srep39737-s1.pdf]

An Adaptation To Life In Acid Through A Novel Mevalonate Pathway.

Jeffrey M. Vinokur,[+,1] Matthew C. Cummins,[+,1] Tyler P. Korman,[1] James U. Bowie[1,*]

SUPPLEMENTARY MATERIALS

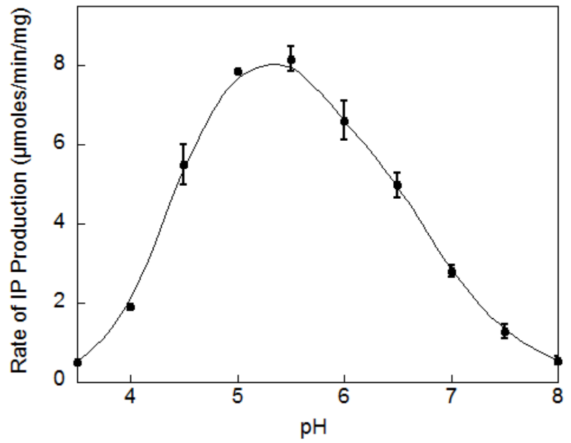

Figure S1. Optimum pH of *P. torridus* MBD. *P. torridus* MBD was assayed from pH 3.5 – 8 in 0.5 unit increments at 60°C.

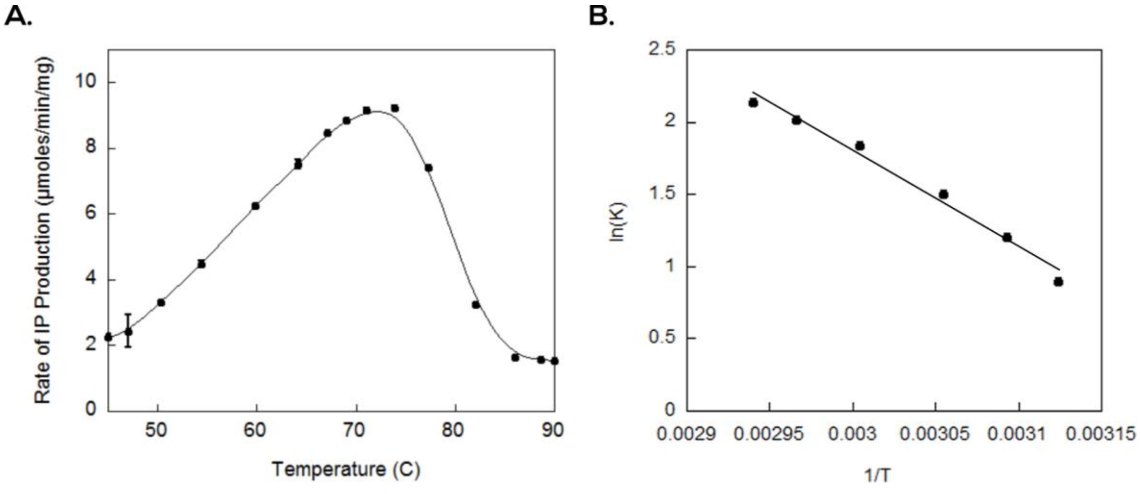

Figure S2. Temperature dependence of *P. torridus* MBD activity. (A.) *P. torridus* MBD was assayed from 45-90°C with the pH held constant at 5.5. (B.) An Arrhenius plot was generated from the data points from 47-67°C by plotting ln(K) vs. 1/T.

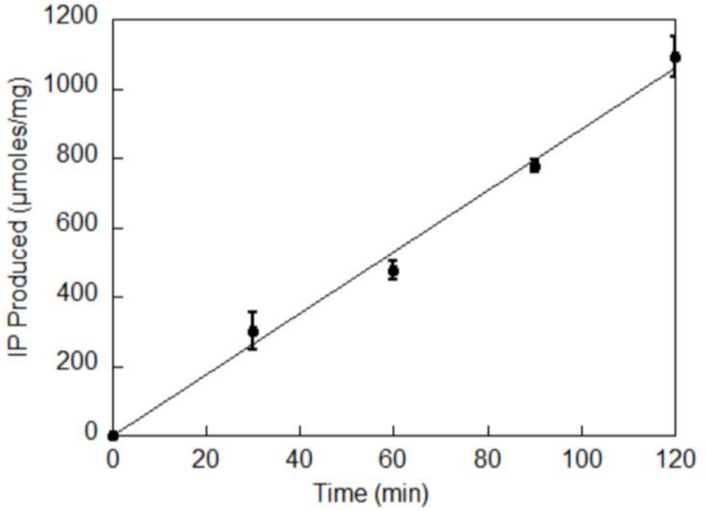

Figure S3. Time course of IP production by *P. torridus* MBD. *P. torridus* MBD was incubated with mevalonate 3,5-bisphosphate at 60°C and pH 5.5. The reaction was stopped at 0, 30, 60, 90, and 120 min. The IP produced by MBD was quantified via our GC-FID assay.
